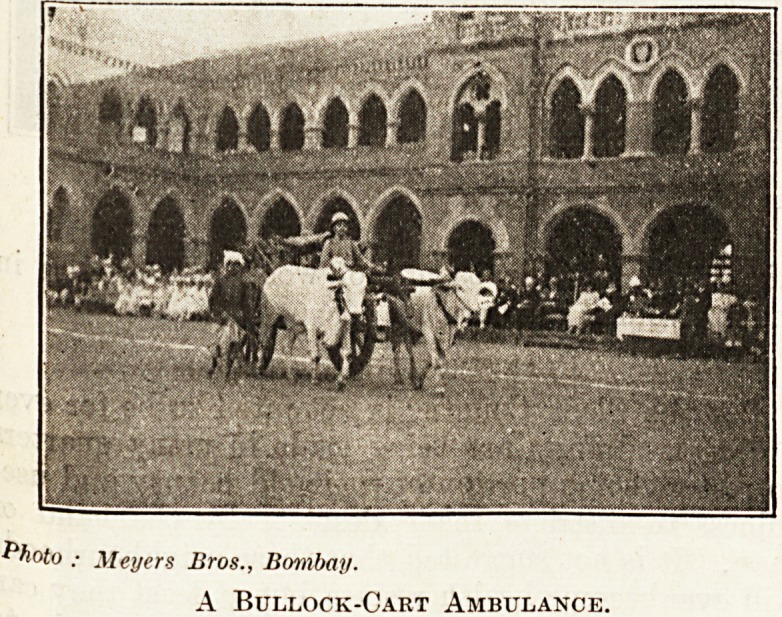# Ambulance Work in Bombay

**Published:** 1923-04

**Authors:** 


### AMBULANCE WORK IN BOMBAY.

THE record of the work done in Bombay by the St. John Ambulance Association is exceedingly creditable. During his eighteen years connection with the Association Mr. D. F. Panthaki, Class Organiser in Bombay, has instructed close on four thousand persons (free of cost), and hundreds of certificates, medallions, labels and pendants have

*Photo : Meyers Bros., Bombay.*

Lady Lloyd Presenting Prizes to a Parsee Division.

been awarded as a result. Those attending the classes have included lawyers, merchants, tradesmen, and boy scouts, and many have passed into the St. John Ambulance Brigade. The past year's work includes 14 classes in First Aid and home nursing, and 187 certificates, &c., were granted. Mrs. Panthaki, who has been working for the Association for eleven years, has, during that time, organised 77 classes in First Aid, home nursing, and home hygiene for women.

*Photo : Meyers Bros., Bombay.*

A Bullock-Cart Ambulance.

¶ The photographs we reproduce were taken at a recent demonstration organised by the Association in Bombay. One photograph shows a bullock cart fitted up as an ambulance ; in the other Lady Lloyd, wife of the Governor of Bombay, is presenting prizes to members of the Parsee Division of the St. John's Ambulance Brigade. Sir George Lloyd, the Governor, is seen wearing a grey top hat.

## Figures and Tables

**Figure f1:**
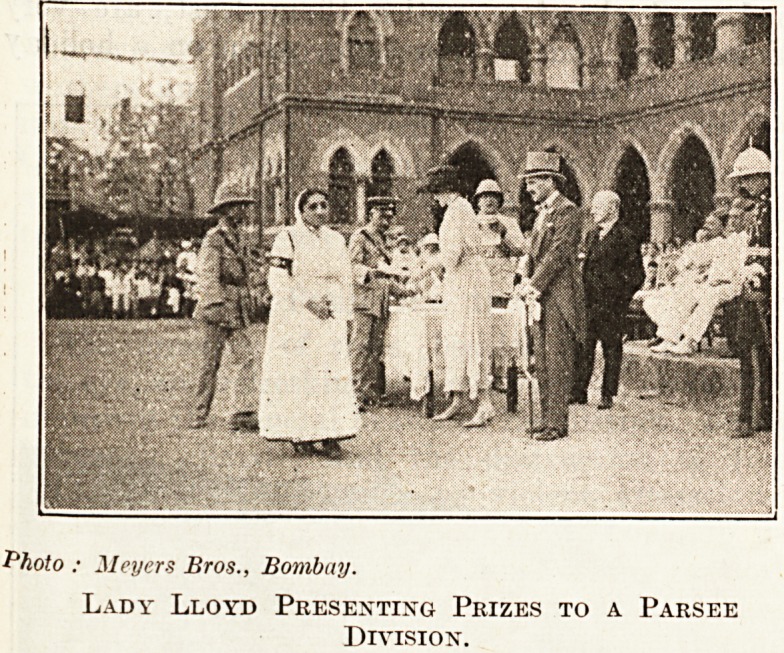


**Figure f2:**